# 1-Methyl-3-*p*-tolyl-3,3a,4,9b-tetra­hydro-1*H*-chromeno[4,3-*c*]isoxazole-3a-carbonitrile

**DOI:** 10.1107/S1600536811021829

**Published:** 2011-06-18

**Authors:** Rajeswari Gangadharan, K. Sethusankar, Gandhi Murugan, Manickam Bakthadoss

**Affiliations:** a Department of Physics, Ethiraj College for Women (Autonomous), Chennai 600 008, India; b Department of Physics, RKM Vivekananda College (Autonomous), Chennai 600 004, India; c Department of Organic Chemistry, University of Madras, Maraimalai Campus, Chennai 600 025, India

## Abstract

In the title compound, C_19_H_18_N_2_O_2_, the dihedral angle between the mean planes of the fused chromeno and isoxazole units is 43.71 (7)°. The isoxazole and pyran rings exhibit envelope and half chair conformations, respectively. The crystal packing is stabilized by inter­molecular C—H⋯π inter­actions.

## Related literature

For uses of chromeno derivatives, see: Carlson (1993 ▶); Sokoloff *et al.* (1990 ▶) and for uses of isoxazole derivatives, see: Kozikowski (1984 ▶); Howe & Shelton (1990 ▶). For a related structure, see: Gangadharan *et al.* (2011 ▶). For puckering parameters, see: Cremer & Pople (1975 ▶). For bond-length and bond-angle distortions, see: Rybarczyk-Pirek *et al.* (2002 ▶); Allen *et al.* (1987 ▶); Raju *et al.* (2002 ▶); For the synthesis of isoxazolidines, see: Bakthadoss & Murugan (2010 ▶).
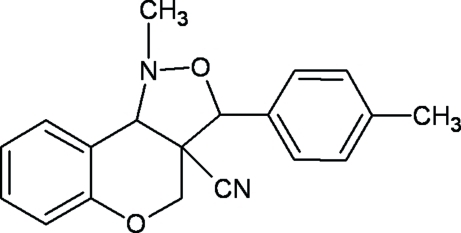


## Experimental

### 

#### Crystal data

C_19_H_18_N_2_O_2_
*M* *_r_* = 306.35
Monoclinic, 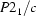

*a* = 8.5344 (3) Å
*b* = 7.6980 (3) Å
*c* = 24.6017 (8) Å
β = 98.234 (2)°
*V* = 1599.62 (10) Å^3^
*Z* = 4
Mo *K*α radiation
μ = 0.08 mm^−1^
*T* = 295 K
0.30 × 0.25 × 0.25 mm

#### Data collection

Bruker Kappa APEXII CCD diffractometer
16796 measured reflections
3606 independent reflections
2571 reflections with *I* > 2σ(*I*)
*R*_int_ = 0.030

#### Refinement

*R*[*F* ^2^ > 2σ(*F* ^2^)] = 0.042
*wR*(*F* ^2^) = 0.118
*S* = 1.04
3606 reflections
210 parameters
H-atom parameters constrained
Δρ_max_ = 0.16 e Å^−3^
Δρ_min_ = −0.14 e Å^−3^

### 

Data collection: *APEX2* (Bruker, 2004 ▶); cell refinement: *SAINT* (Bruker, 2004 ▶); data reduction: *SAINT*; program(s) used to solve structure: *SHELXS97* (Sheldrick, 2008 ▶); program(s) used to refine structure: *SHELXL97* (Sheldrick, 2008 ▶); molecular graphics: *ORTEP-3* (Farrugia, 1997 ▶); software used to prepare material for publication: *SHELXL97* and *PLATON* (Spek, 2009 ▶).

## Supplementary Material

Crystal structure: contains datablock(s) global, I. DOI: 10.1107/S1600536811021829/rk2276sup1.cif

Structure factors: contains datablock(s) I. DOI: 10.1107/S1600536811021829/rk2276Isup2.hkl

Supplementary material file. DOI: 10.1107/S1600536811021829/rk2276Isup3.cml

Additional supplementary materials:  crystallographic information; 3D view; checkCIF report

## Figures and Tables

**Table 1:** Hydrogen-bond geometry (Å, °)

*D*—H⋯*A*	*D*—H	H⋯*A*	*D*⋯*A*	*D*—H⋯*A*
C3—H3⋯*Cg*3^i^	0.93	2.99	3.8075 (18)	147

Symmetry code: (i) 
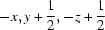
.

**Fig. 1.**
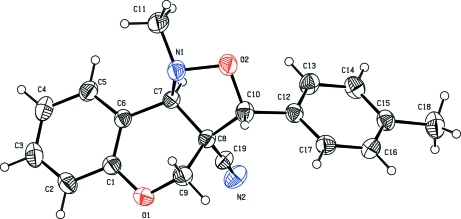
The molecular structure of the title compound with the atom numbering scheme. Displacement ellipsoids are drawn at 30% probability level. H atoms are presented as small spheres of arbitary radius.

**Fig. 2.**
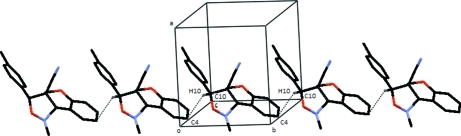
Formation of C—H···C intermolecular bonding in the title compound.

## supplementary crystallographic information

### Comment

Chromenopyrroles are used in the treatment of Parkinsons disease (Carlson, 1993) and memory disorders(Sokoloff *et al.*, 1990). Isoxazoline derivatives have been shown to be efficient precursors for many synthetic intermediates including γ-amino alchols and β-hydroxy ketones (Kozikowski, 1984). Spiroisoxazolines display interesting biological properties such as herbicidal, plant growth regulators and antitumour activities (Howe & Shelton, 1990). These observations prompted us to synthesize the title compound with fused chromeno and isoxazole rings (Bakthadoss & Murugan, 2010).

In the title molecule (Fig 1), the fused benzene and pyran rings forming the chromeno system are inclined to one another at a dihedral angle of 4.47 (7)° between the best planes of the rings. The six membered pyran ring adopts a *half chair* conformation with puckering amplitude Q= 0.4782 (15)Å, θ = 50.93 (17)° and φ = 278.3 (2)° (Cremer & Pople, 1975). In the pyran ring the C—C bond distances vary from a minimum of 1.3901 (19)Å to a maximum of 1.5332 (19)Å in comparison with a typical aromatic bond length of 1.384 (13)Å (Allen *et al.*, 1987). This could be attributed to the presence of the heteroatom O1 in the cyclic system and also to the fusion of the pyran and isoxazole ring systems (Rybarczyk-Pirek *et al.*, 2002).

The fusion between the isoxazole and the pyran rings at C7 and C8 is in *cis*-form. The dihedral angle between the fused chromeno and the isoxazole moieties is 43.71 (7)°.

The isoxazole ring adopts an *envelope* conformation at N1 with puckering parameters q2 = 0.5179 (14)Å and φ2 = 217.11 (16)° (Cremer & Pople,1975). In the isoxazole ring, enlargement of bond lengths and bond angles are observed at the points of linkages of substituents and fusion to the pyran ring (Raju *et al.*, 2002).

The phenyl ring (C12–C17) substituent is almost perpendicular to the five membered isoxazole ring, the dihedral angle between them being 81.26 (8)°. The geometric parameters of the title compound agree well with reported structure (Gangadharan *et al.*, 2011).

The crystal packing is stabilized by C—H···C and C—H···π interactions (C3—H···Cg3, where Cg3 is the centroid of the six membered ring defined by atoms C1–C6). The symmetry codes are: (i) *x*, *y*-1, *z*; (ii) -*x*, 1/2+*y*, 1/2-*z*. The packing view of the title compound shown in Fig. 2.

### Experimental

A mixture of compound (*E*)-2-((2-formylphenoxy)methyl)-3-*p*-tolylacylonitrile (1 mmol) with *N*-methylhydroxylamine hydrochloride (1.1 mmol), pyridine (0.24 ml,3 mmol) and ethanol (5 ml) were placed in a round bottom flask and refluxed for 6 h. After completion of the reaction as indicated by *TLC*, the reaction mixture was concentrated under reduced pressure. The crude product was diluted with water (10 ml), dilute HCl (5 ml) and extracted with ethylacetate (20 ml). The organic layer was washed with brine solution (10 ml) and concentrated. The crude product was purified by column chromatography to provide the pure desired product as colourless solid.

### Refinement

All hydrogen atoms were placed in calculated positions with C—H = 0.93–0.98Å and refined in riding model with isotropic displacement parameters: *U*_iso_(H) = 1.5*U*_eq_(C) for methyl group and *U*_iso_(H)=1.2*U*_eq_(C) for other groups.

### Crystal data

**Table:**

C_19_H_18_N_2_O_2_	*F*(000) = 648
*M**_r_* = 306.35	*D*_x_ = 1.272 Mg m^−^^3^
Monoclinic, *P*2_1_/*c*	Mo *K*α radiation, λ = 0.71073 Å
Hall symbol: -P 2ybc	Cell parameters from 3606 reflections
*a* = 8.5344 (3) Å	θ = 1.0–27.4°
*b* = 7.6980 (3) Å	µ = 0.08 mm^−^^1^
*c* = 24.6017 (8) Å	*T* = 295 K
β = 98.234 (2)°	Block, colourless
*V* = 1599.62 (10) Å^3^	0.30 × 0.25 × 0.25 mm
*Z* = 4	

### Data collection

**Table:**

Bruker Kappa APEXII CCD diffractometer	2571 reflections with *I* > 2σ(*I*)
Radiation source: fine-focus sealed tube	*R*_int_ = 0.030
graphite	θ_max_ = 27.4°, θ_min_ = 2.4°
ω–scans	*h* = −10→11
16796 measured reflections	*k* = −9→9
3606 independent reflections	*l* = −31→31

### Refinement

**Table:**

Refinement on *F*^2^	Primary atom site location: structure-invariant direct methods
Least-squares matrix: full	Secondary atom site location: difference Fourier map
*R*[*F*^2^ > 2σ(*F*^2^)] = 0.042	Hydrogen site location: inferred from neighbouring sites
*wR*(*F*^2^) = 0.118	H-atom parameters constrained
*S* = 1.04	*w* = 1/[σ^2^(*F*_o_^2^) + (0.0533*P*)^2^ + 0.223*P*] where *P* = (*F*_o_^2^ + 2*F*_c_^2^)/3
3606 reflections	(Δ/σ)_max_ < 0.001
210 parameters	Δρ_max_ = 0.16 e Å^−^^3^
0 restraints	Δρ_min_ = −0.14 e Å^−^^3^

### Special details

**Table:**

Geometry. All s.u.'s (except the s.u. in the dihedral angle between two l.s. planes) are estimated using the full covariance matrix. The cell s.u.'s are taken into account individually in the estimation of s.u.'s in distances, angles and torsion angles; correlations between s.u.'s in cell parameters are only used when they are defined by crystal symmetry. An approximate (isotropic) treatment of cell s.u.'s is used for estimating s.u.'s involving l.s. planes.
Refinement. Refinement of *F*^2^ against ALL reflections. The weighted *R*-factor *wR* and goodness of fit *S* are based on *F*^2^, conventional *R*-factors *R* are based on *F*, with *F* set to zero for negative *F*^2^. The threshold expression of *F*^2^ > σ(*F*^2^) is used only for calculating *R*-factors(gt) *etc*. and is not relevant to the choice of reflections for refinement. *R*-factors based on *F*^2^ are statistically about twice as large as those based on *F*, and *R*-factors based on ALL data will be even larger.

### Fractional atomic coordinates and isotropic or equivalent isotropic displacement parameters (Å^2^)

**Table:**

	*x*	*y*	*z*	*U*_iso_*/*U*_eq_	
C1	0.22956 (16)	0.74379 (19)	0.22503 (6)	0.0473 (3)	
C2	0.20315 (19)	0.8782 (2)	0.25995 (6)	0.0580 (4)	
H2	0.2518	0.8771	0.2963	0.070*	
C3	0.1058 (2)	1.0123 (2)	0.24110 (8)	0.0655 (5)	
H3	0.0882	1.1022	0.2647	0.079*	
C4	0.0333 (2)	1.0157 (2)	0.18741 (8)	0.0661 (5)	
H4	−0.0330	1.1073	0.1747	0.079*	
C5	0.05999 (17)	0.8817 (2)	0.15272 (7)	0.0559 (4)	
H5	0.0123	0.8851	0.1163	0.067*	
C6	0.15635 (15)	0.74186 (18)	0.17079 (5)	0.0436 (3)	
C7	0.19011 (15)	0.60029 (18)	0.13206 (5)	0.0437 (3)	
H7	0.2107	0.6513	0.0973	0.052*	
C8	0.33071 (16)	0.48679 (18)	0.15615 (5)	0.0442 (3)	
C9	0.32444 (19)	0.4600 (2)	0.21731 (6)	0.0540 (4)	
H9A	0.2260	0.4026	0.2219	0.065*	
H9B	0.4110	0.3849	0.2326	0.065*	
C10	0.29378 (17)	0.3102 (2)	0.12512 (6)	0.0527 (4)	
H10	0.2751	0.2212	0.1519	0.063*	
C11	−0.07140 (19)	0.5205 (3)	0.08314 (8)	0.0816 (6)	
H11A	−0.0377	0.5647	0.0502	0.122*	
H11B	−0.1272	0.6096	0.0998	0.122*	
H11C	−0.1402	0.4227	0.0743	0.122*	
C12	0.41716 (17)	0.24478 (18)	0.09254 (6)	0.0474 (3)	
C13	0.43199 (19)	0.3076 (2)	0.04099 (6)	0.0570 (4)	
H13	0.3610	0.3903	0.0246	0.068*	
C14	0.5515 (2)	0.2484 (2)	0.01362 (6)	0.0594 (4)	
H14	0.5591	0.2913	−0.0212	0.071*	
C15	0.65985 (18)	0.1273 (2)	0.03664 (6)	0.0540 (4)	
C16	0.64196 (19)	0.0627 (2)	0.08744 (7)	0.0602 (4)	
H16	0.7124	−0.0210	0.1036	0.072*	
C17	0.52204 (19)	0.1192 (2)	0.11499 (6)	0.0564 (4)	
H17	0.5117	0.0722	0.1491	0.068*	
C18	0.7938 (2)	0.0675 (3)	0.00727 (8)	0.0795 (6)	
H18A	0.7638	0.0806	−0.0316	0.119*	
H18B	0.8165	−0.0525	0.0157	0.119*	
H18C	0.8862	0.1362	0.0191	0.119*	
C19	0.47926 (17)	0.57010 (19)	0.14768 (6)	0.0469 (3)	
N1	0.06659 (14)	0.46627 (17)	0.12132 (5)	0.0582 (4)	
N2	0.59187 (16)	0.64169 (19)	0.14207 (6)	0.0678 (4)	
O1	0.33511 (13)	0.61910 (14)	0.24647 (4)	0.0597 (3)	
O2	0.14917 (12)	0.34224 (16)	0.08996 (5)	0.0686 (3)	

### Atomic displacement parameters (Å^2^)

**Table:**

	*U*^11^	*U*^22^	*U*^33^	*U*^12^	*U*^13^	*U*^23^
C1	0.0414 (8)	0.0510 (8)	0.0507 (8)	−0.0054 (6)	0.0103 (6)	−0.0034 (6)
C2	0.0541 (9)	0.0643 (10)	0.0576 (9)	−0.0120 (8)	0.0154 (7)	−0.0173 (8)
C3	0.0567 (10)	0.0600 (10)	0.0846 (12)	−0.0077 (8)	0.0269 (9)	−0.0238 (9)
C4	0.0535 (9)	0.0552 (10)	0.0924 (13)	0.0099 (8)	0.0198 (9)	−0.0044 (9)
C5	0.0453 (8)	0.0591 (10)	0.0635 (9)	0.0059 (7)	0.0086 (7)	−0.0008 (7)
C6	0.0345 (7)	0.0484 (8)	0.0494 (7)	−0.0031 (6)	0.0114 (5)	−0.0031 (6)
C7	0.0363 (7)	0.0498 (8)	0.0451 (7)	0.0003 (6)	0.0063 (5)	−0.0022 (6)
C8	0.0403 (7)	0.0442 (8)	0.0485 (7)	0.0010 (6)	0.0077 (6)	−0.0006 (6)
C9	0.0603 (9)	0.0506 (9)	0.0511 (8)	0.0042 (7)	0.0078 (7)	0.0046 (7)
C10	0.0504 (9)	0.0462 (8)	0.0630 (9)	−0.0033 (7)	0.0137 (7)	−0.0057 (7)
C11	0.0406 (9)	0.1028 (15)	0.0972 (13)	0.0017 (9)	−0.0049 (8)	−0.0387 (12)
C12	0.0488 (8)	0.0407 (8)	0.0528 (8)	−0.0017 (6)	0.0076 (6)	−0.0052 (6)
C13	0.0638 (10)	0.0455 (8)	0.0614 (9)	0.0078 (7)	0.0084 (7)	0.0074 (7)
C14	0.0741 (11)	0.0536 (9)	0.0529 (8)	−0.0037 (8)	0.0173 (8)	0.0015 (7)
C15	0.0541 (9)	0.0487 (9)	0.0603 (9)	−0.0047 (7)	0.0122 (7)	−0.0128 (7)
C16	0.0563 (9)	0.0580 (10)	0.0644 (10)	0.0141 (8)	0.0026 (7)	−0.0008 (8)
C17	0.0637 (10)	0.0576 (9)	0.0474 (8)	0.0064 (8)	0.0057 (7)	0.0036 (7)
C18	0.0744 (12)	0.0772 (13)	0.0927 (13)	−0.0026 (10)	0.0327 (10)	−0.0224 (10)
C19	0.0395 (8)	0.0453 (8)	0.0549 (8)	0.0063 (7)	0.0038 (6)	0.0034 (6)
N1	0.0395 (7)	0.0621 (8)	0.0736 (8)	−0.0039 (6)	0.0095 (6)	−0.0215 (7)
N2	0.0468 (8)	0.0640 (9)	0.0923 (10)	−0.0020 (7)	0.0093 (7)	0.0111 (8)
O1	0.0649 (7)	0.0651 (7)	0.0462 (5)	0.0059 (6)	−0.0022 (5)	−0.0047 (5)
O2	0.0461 (6)	0.0732 (8)	0.0851 (8)	−0.0001 (6)	0.0041 (5)	−0.0351 (6)

### Geometric parameters (Å, °)

**Table:**

C1—O1	1.3692 (18)	C10—C12	1.4989 (19)
C1—C2	1.384 (2)	C10—H10	0.9800
C1—C6	1.3901 (19)	C11—N1	1.458 (2)
C2—C3	1.364 (2)	C11—H11A	0.9600
C2—H2	0.9300	C11—H11B	0.9600
C3—C4	1.376 (2)	C11—H11C	0.9600
C3—H3	0.9300	C12—C17	1.378 (2)
C4—C5	1.378 (2)	C12—C13	1.380 (2)
C4—H4	0.9300	C13—C14	1.378 (2)
C5—C6	1.389 (2)	C13—H13	0.9300
C5—H5	0.9300	C14—C15	1.377 (2)
C6—C7	1.5025 (19)	C14—H14	0.9300
C7—N1	1.4716 (18)	C15—C16	1.374 (2)
C7—C8	1.5332 (19)	C15—C18	1.509 (2)
C7—H7	0.9800	C16—C17	1.376 (2)
C8—C19	1.462 (2)	C16—H16	0.9300
C8—C9	1.5272 (19)	C17—H17	0.9300
C8—C10	1.569 (2)	C18—H18A	0.9600
C9—O1	1.4155 (18)	C18—H18B	0.9600
C9—H9A	0.9700	C18—H18C	0.9600
C9—H9B	0.9700	C19—N2	1.1333 (18)
C10—O2	1.4235 (18)	N1—O2	1.4694 (16)
			
O1—C1—C2	116.22 (13)	O2—C10—H10	108.6
O1—C1—C6	122.86 (13)	C12—C10—H10	108.6
C2—C1—C6	120.87 (14)	C8—C10—H10	108.6
C3—C2—C1	120.02 (15)	N1—C11—H11A	109.5
C3—C2—H2	120.0	N1—C11—H11B	109.5
C1—C2—H2	120.0	H11A—C11—H11B	109.5
C2—C3—C4	120.58 (15)	N1—C11—H11C	109.5
C2—C3—H3	119.7	H11A—C11—H11C	109.5
C4—C3—H3	119.7	H11B—C11—H11C	109.5
C3—C4—C5	119.26 (16)	C17—C12—C13	118.32 (14)
C3—C4—H4	120.4	C17—C12—C10	119.16 (13)
C5—C4—H4	120.4	C13—C12—C10	122.51 (14)
C4—C5—C6	121.65 (15)	C14—C13—C12	120.32 (15)
C4—C5—H5	119.2	C14—C13—H13	119.8
C6—C5—H5	119.2	C12—C13—H13	119.8
C5—C6—C1	117.59 (13)	C15—C14—C13	121.60 (14)
C5—C6—C7	121.19 (12)	C15—C14—H14	119.2
C1—C6—C7	121.06 (13)	C13—C14—H14	119.2
N1—C7—C6	115.14 (11)	C16—C15—C14	117.62 (14)
N1—C7—C8	99.83 (11)	C16—C15—C18	121.05 (16)
C6—C7—C8	112.25 (11)	C14—C15—C18	121.33 (16)
N1—C7—H7	109.7	C15—C16—C17	121.40 (15)
C6—C7—H7	109.7	C15—C16—H16	119.3
C8—C7—H7	109.7	C17—C16—H16	119.3
C19—C8—C9	110.68 (12)	C16—C17—C12	120.70 (14)
C19—C8—C7	109.97 (11)	C16—C17—H17	119.7
C9—C8—C7	108.78 (11)	C12—C17—H17	119.7
C19—C8—C10	115.37 (12)	C15—C18—H18A	109.5
C9—C8—C10	109.25 (12)	C15—C18—H18B	109.5
C7—C8—C10	102.36 (11)	H18A—C18—H18B	109.5
O1—C9—C8	111.94 (12)	C15—C18—H18C	109.5
O1—C9—H9A	109.2	H18A—C18—H18C	109.5
C8—C9—H9A	109.2	H18B—C18—H18C	109.5
O1—C9—H9B	109.2	N2—C19—C8	176.76 (16)
C8—C9—H9B	109.2	C11—N1—O2	104.57 (12)
H9A—C9—H9B	107.9	C11—N1—C7	114.00 (14)
O2—C10—C12	110.34 (12)	O2—N1—C7	99.48 (10)
O2—C10—C8	104.01 (11)	C1—O1—C9	114.86 (11)
C12—C10—C8	116.36 (12)	C10—O2—N1	103.46 (10)
			
O1—C1—C2—C3	176.76 (13)	C9—C8—C10—C12	−123.06 (14)
C6—C1—C2—C3	−0.8 (2)	C7—C8—C10—C12	121.75 (13)
C1—C2—C3—C4	−0.1 (2)	O2—C10—C12—C17	−142.83 (14)
C2—C3—C4—C5	0.0 (2)	C8—C10—C12—C17	99.01 (16)
C3—C4—C5—C6	1.0 (2)	O2—C10—C12—C13	38.3 (2)
C4—C5—C6—C1	−1.9 (2)	C8—C10—C12—C13	−79.82 (18)
C4—C5—C6—C7	−177.41 (14)	C17—C12—C13—C14	−1.5 (2)
O1—C1—C6—C5	−175.61 (13)	C10—C12—C13—C14	177.30 (14)
C2—C1—C6—C5	1.8 (2)	C12—C13—C14—C15	−0.6 (3)
O1—C1—C6—C7	−0.1 (2)	C13—C14—C15—C16	2.0 (2)
C2—C1—C6—C7	177.32 (13)	C13—C14—C15—C18	−177.88 (15)
C5—C6—C7—N1	−81.82 (17)	C14—C15—C16—C17	−1.3 (2)
C1—C6—C7—N1	102.86 (15)	C18—C15—C16—C17	178.64 (16)
C5—C6—C7—C8	164.86 (13)	C15—C16—C17—C12	−0.9 (3)
C1—C6—C7—C8	−10.46 (18)	C13—C12—C17—C16	2.3 (2)
N1—C7—C8—C19	154.67 (11)	C10—C12—C17—C16	−176.59 (14)
C6—C7—C8—C19	−82.87 (14)	C6—C7—N1—C11	77.26 (16)
N1—C7—C8—C9	−83.97 (13)	C8—C7—N1—C11	−162.35 (12)
C6—C7—C8—C9	38.50 (15)	C6—C7—N1—O2	−172.04 (11)
N1—C7—C8—C10	31.56 (12)	C8—C7—N1—O2	−51.65 (12)
C6—C7—C8—C10	154.02 (11)	C2—C1—O1—C9	161.37 (13)
C19—C8—C9—O1	60.05 (16)	C6—C1—O1—C9	−21.07 (19)
C7—C8—C9—O1	−60.88 (15)	C8—C9—O1—C1	52.08 (17)
C10—C8—C9—O1	−171.86 (12)	C12—C10—O2—N1	−157.88 (11)
C19—C8—C10—O2	−119.20 (13)	C8—C10—O2—N1	−32.38 (14)
C9—C8—C10—O2	115.38 (13)	C11—N1—O2—C10	172.10 (14)
C7—C8—C10—O2	0.19 (14)	C7—N1—O2—C10	54.09 (13)
C19—C8—C10—C12	2.36 (18)		

### Hydrogen-bond geometry (Å, °)

**Table:**

Cg3 is the centroid of the C1–C6 ring.

**Table:**

*D*—H···*A*	*D*—H	H···*A*	*D*···*A*	*D*—H···*A*
C10—H10···C4^i^	0.98	2.84	3.662 (2)	143
C3—H3···Cg3^ii^	0.93	2.99	3.8075 (18)	147

Symmetry codes: (i) *x*, *y*−1, *z*; (ii) −*x*, *y*+1/2, −*z*+1/2.
